# Antimicrobial Carvacrol-Containing Polypropylene Films: Composition, Structure and Function

**DOI:** 10.3390/polym10010079

**Published:** 2018-01-16

**Authors:** Max Krepker, Ofer Prinz-Setter, Rotem Shemesh, Anita Vaxman, David Alperstein, Ester Segal

**Affiliations:** 1 Department of Biotechnology and Food Engineering, Technion—Israel Institute of Technology, Haifa 3200003, Israel; maks@campus.technion.ac.il (M.K.); oferp@campus.technion.ac.il (O.P.-S.); 2 Carmel Olefins Ltd., P.O. Box 1468, Haifa 31014, Israel; Srotem@bazan.co.il (R.S.); Vaxman.anita@gmail.com (A.V.); 3 Department of Mechanical Engineering, Ort Braude College, P.O. Box 78, Karmiel 2161002, Israel; davida@braude.ac.il; 4 The Russell Berrie Nanotechnology Institute, Technion—Israel Institute of Technology, Haifa 3200003, Israel

**Keywords:** antimicrobial, polypropylene, halloysite nanotubes, carvacrol, essential oils

## Abstract

Significant research has been directed toward the incorporation of bioactive plant extracts or essential oils (EOs) into polymers to endow the latter with antimicrobial functionality. EOs offer a unique combination of having broad antimicrobial activity from a natural source, generally recognized as safe (GRAS) recognition in the US, and a volatile nature. However, their volatility also presents a major challenge in their incorporation into polymers by conventional high-temperature-processing techniques. Herein, antimicrobial polypropylene (PP) cast films were produced by incorporating carvacrol (a model EO) or carvacrol, loaded into halloysite nanotubes (HNTs), via melt compounding. We studied the composition-structure-property relationships in these systems, focusing on the effect of carvacrol on the composition of the films, the PP crystalline phase and its morphology and the films’ mechanical and antimicrobial properties. For the first time, molecular dynamics simulations were applied to reveal the complex interactions between the components of these carvacrol-containing systems. We show that strong molecular interactions between PP and carvacrol minimize the loss of this highly-volatile EO during high-temperature polymer processing, enabling semi-industrial scale production. The resulting films exhibit outstanding antimicrobial properties against model microorganisms (*Escherichia coli* and *Alternaria alternata*). The PP/(HNTs-carvacrol) nanocomposite films, containing the carvacrol-loaded HNTs, display a higher level of crystalline order, superior mechanical properties and prolonged release of carvacrol, in comparison to PP/carvacrol blends. These properties are ascribed to the role of HNTs in these nanocomposites and their effect on the PP matrix and retained carvacrol content.

## 1. Introduction

Colonization of microorganisms on polymeric surfaces is a major concern, as it may lead to severe contamination and biofilm formation. These contaminations, caused by pathogenic bacteria and fungi, pose an acute challenge in the field of medical devices, healthcare and hygienic applications, food packaging and in industrial processing environments [1]. The use of natural or synthetically-derived antimicrobial polymers is a possible approach to combat these contaminations [2]. Yet, incorporation of low-molecular weight antimicrobials into polymers is one of the most straight-forward schemes to produce antimicrobial polymeric systems. A variety of organic and inorganic antimicrobial compounds, e.g., triclosan or metal ions, were successfully incorporated into different polymers, resulting in materials with long-term antimicrobial activity [3,4]. However, some of these systems exhibit limitations, which are associated mainly to their mode of action (antimicrobial effect is exerted only upon direct contact with the target microorganism) as well as safety concerns regarding their usage [5]. Thus, naturally-derived compounds, such as bacteriocins, enzymes and volatile essential oils, have emerged as attractive antimicrobials, owing to their diverse functionally and potential consumer acceptance [6,7]. Essential oils (EOs), which are natural and highly-effective antimicrobials against both bacteria and fungi, and are categorized as GRAS (generally recognized as safe) by the Food and Drug Administration (FDA) [8,9,10,11]. Their incorporation into polymers offers significant advantages, as they can be released as a vapor, exerting an antimicrobial effect, both on the polymer surface and in its proximity [12,13,14]. Yet, the integration of these sensitive compounds with commodity polymers using conventional manufacturing techniques is challenging, due to their loss during high-temperature processing and diminished antimicrobial efficacy [15,16,17,18,19]. Moreover, the incorporation of such low-molecular weight additives, which act as plasticizers, may profoundly affect the physico-mechanical properties of polymers [6].

To answer these challenges, we and others [20,21,22] have shown that organoclays can be used as active carriers for EOs, to protect them during the high-temperature compounding step with different polymers, while preserving their antimicrobial properties. The organo-modification of clay was found to be crucial for intercalation of model EO molecules (carvacrol) in between silicate galleries, which, in turn significantly enhance the thermal stability of carvacrol [20]. The resulting nanocomposites (based on low-density polyethylene, LDPE) exhibited significantly higher carvacrol content in the film, combined with slower out-diffusion of the carvacrol molecules, in comparison to LDPE/carvacrol films as well as superior and prolonged antibacterial activity against *Escherichia coli* (*E. coli*) and *Listeria innocua* [21]. Recently, Halloysite nanotubes (HNTs), which are naturally-occurring clays with a characteristic tubular structure and chemical composition similar to kaolin, have emerged as promising nanomaterials for the encapsulation of different active molecules [23,24,25,26,27,28,29]. In addition, the combination of their low cost, excellent mechanical properties, high thermal stability and unique nanostructure, has attracted significant attention in the field of high-performance polymer nanocomposites for structural and functional applications [30,31,32,33].

In our recent studies, we have demonstrated that HNTs can be used as active carriers for carvacrol and for synergistic mixture of carvacrol and thymol in LDPE [34,35] and polyamide (PA) [36], processed at elevated temperatures (up to 250 °C). HNTs were found to exhibit improved dispersibility in polymers and enhance the thermal stability of the encapsulated EOs, in comparison to organoclays. Thus, they allowed the production of EOs-containing nanocomposite systems with high EO content and a sustained release profile of volatile compounds. Moreover, the resulting films displayed excellent and extended antibacterial, antibiofilm and antifungal properties, both in synthetic media and in complex food systems [34,35,36].

In this work, we aim to extend this approach to polypropylene (PP), which is widely used in industry owing to its well-balanced physical and mechanical properties, easy processability and low cost. Several studies have reported different strategies to integrate neat EOs with PP by coating formulations [37,38,39,40] and by high-temperature processing [16]. We loaded carvacrol into HNTs, in a pre-compounding step, resulting in HNTs-carvacrol hybrids. The latter were melt-compounded with PP and films were produced by cast extrusion. We systematically investigated the composition-structure-property relationships of these nanocomposites. To reveal the complex interactions between the components of these ternary PP-HNTs-carvacrol systems, we applied molecular dynamics simulations and correlated the results with the role of HNTs in these nanocomposites and their effect on the retained carvacrol. Specifically, we characterized the carvacrol concentration in the final films, its release profile and the antimicrobial activity of the films. Further, we thoroughly characterized the crystalline and amorphous phases of PP in the nanocomposites and in the reference films (i.e., neat PP, PP/HNTs and PP/carvacrol) and compared the effects of the HNTs and carvacrol on physical and mechanical properties of the polymer.

## 2. Materials and Methods

### 2.1. Materials

Halloysite nanotubes (HNTs) were supplied by NaturalNano (Rochester, NY, USA). Isotactic polypropylene homopolymer (PP) with a melt flow index (MFI) value of 2.5–3.5 g (10 min^−1^), Capilene™, was supplied by Carmel Olefins Ltd. (Haifa, Israel). Carvacrol (1-methyl-4-(1-methylethylidene)cyclohexene, >97%, CAS 586-62-9), yeast extract and tryptone for lysogeny broth (LB) medium, NaCl, Bacto agar and potato dextrose agar (PDA) were purchased from Sigma–Aldrich (Rehovot, Israel).

### 2.2. Preparation of HNTs Loaded with Carvacrol

Halloysite nanotubes (HNTs) were dried at 150 °C for 3 h prior to use, to remove adsorbed moisture. The dried HNTs were shear-mixed with carvacrol, followed by ultrasonication (Vibra cell VCX 750 instrument, Sonics & Materials Inc., Newtown, CT, USA) at a constant amplitude of 40% for 20 min in an ice bath [35,36]. SAFETY NOTATION: This procedure should be performed in a fume hood to minimize inhalation of HNTs [41].

### 2.3. Preparation of PP Films

Polypropylene was melt-compounded at 190 °C with neat HNTs, pure carvacrol and HNTs-carvacrol hybrids, using a 25-mm twin-screw extruder (Berstorff, Munich, Germany) with a length/diameter (L/D) ratio of 25:1 at a screw speed of 300 rpm and feeding rate of 5 kg·h^−1^. Following the melt-compounding process, ~100 µm thick films were prepared by cast extrusion, using a 45-mm screw diameter extruder (Dr. Collin GmbH, Ebersberg, Germany) at 180 °C. Neat polypropylene films were prepared under similar conditions. Table 1 specifies the composition of the studied polymer nanocomposites and reference films.

### 2.4. Characterization of Films

#### 2.4.1. High-Resolution Scanning Electron Microscopy

The nanostructure of cross-sectioned PP-based nanocomposite films was studied using a Carl Zeiss Ultra Plus (Zeiss, Oberkochen, Germany) high-resolution scanning electron microscope (HR-SEM), operated at 1 keV accelerating voltage. Films were fractured in liquid nitrogen prior to observation.

#### 2.4.2. Thermal Gravimetric Analysis (TGA)

The post-processing content of carvacrol and thermal stability of the PP matrix were investigated by thermal gravimetric analysis (TGA), using a TGA-Q5000 system (TA Instruments, Newcastle, DE, USA). PP-based films were heated under a nitrogen atmosphere, from room temperature to 600 °C, at a heating rate of 20 °C·min^−1^. The mass loss at 225 °C was attributed to the total volatile content. The results were analyzed using Universal Analysis 200 version 4.5A build 4.5.0.5 software (TA Instruments, Newcastle, DE, USA). The measurements were performed in triplicate.

#### 2.4.3. Infrared Spectroscopy

The PP-based films were characterized by Fourier-transform infrared (FTIR) spectroscopy, in transmission mode, using a Thermo 6700 FTIR instrument and OMNIC v8.0 software (Waltham, MA, USA). Spectra resulted from co-addition of 64 interferograms, at 4 cm^−1^ resolution, between 4000 cm^−1^ to 400 cm^−1^. Data analyses and calculations on the A_841_/A_973_ bands ratio was performed by TQ analyst v8.0 software (Waltham, MA, USA).

#### 2.4.4. Differential Scanning Calorimetry (DSC)

The thermal properties of the resulting films were studied by DSC (DSC 1, Mettler-Toledo, Greifensee, Switzerland). Films were heated from room temperature to 200 °C at a rate of 10 °C·min^−1^ under a nitrogen atmosphere. The PP crystallinity (*c*) was corrected for the presence of additives using Equation (1):

(1) $$c_{i}=\frac{\Delta H_{m}}{f_{p}\Delta H_{m}^{0}},$$

where *i* is the heating cycle number, Δ*H_m_* (J·g^−1^) is the latent heat of fusion of the sample, *f_p_* is the PP weight fraction in the sample, and $\Delta H_{m}^{0}$ is the theoretical latent heat of fusion for 100% crystalline PP, 207 J·g^−1^ [42].

The characterization of glass transition temperature (*T_g_*) was carried out using a DSC8000 (Perkin Elmer, Waltham, MA, USA) system. The specimens were heated from −50 °C to 50 °C, at a rate of 50 °C·min^−1^, under a nitrogen atmosphere. Extrapolated onset temperature, i.e., the point of intersection of the tangent drawn at the point of greatest slope on the transition curve with the extrapolated baseline prior to the transition, is reported as the *T_g_*, according to the ASTM E1356 standard procedure [43].

#### 2.4.5. Wide-Angle X-ray (WAXS) Diffraction

Wide-angle X-ray (WAXS) diffraction spectra were collected using a Rigaku X-ray diffractometer (SmartLab, Rigaku, Tokyo, Japan), in the range between 5° and 30°. The CuKα radiation source was operated at 40 kV power and 30 mA current. The amorphous halos and crystalline reflections were fitted using Lorentzian functions, using PDXL 2 software version 2.6.1.2 (Rigaku Corporation, Tokyo, Japan). The degree of crystallinity was calculated as *I_c_*/(*I_c_* + *I_a_*), where *I_c_* is the diffracted intensity from all resolved crystalline reflections and *I_a_* is the diffraction intensity under the amorphous halo.

#### 2.4.6. Carvacrol Release Studies

The accelerated release of carvacrol from the films was studied by isothermal gravimetric analysis, using a TGA-Q5000 system (TA Instruments, Newcastle, DE, USA), at a constant temperature of 60 °C, under a nitrogen atmosphere. A film sample (7 mm in diameter) was placed in a platinum pan and was held at 60 °C until a constant mass was attained. The sample’s mass loss at this temperature was attributed to the release of carvacrol.

Several methods have been reported to characterize the diffusion of active agents and other additives in polymers [44,45,46,47,48]. In this study, we express the diffusion coefficient from the initial linear slope of the fractional mass release ratio vs. $t^{1/2}$ according to Equation (2) [44,48].

(2) $$\frac{m_{t}}{m_{\infty }}=4{\left(\frac{Dt}{\pi l^{2}}\right)}^{1/2},$$

where $m_{t}$ and $m_{\infty }$ are the amounts of additive (carvacrol) released from the film at time, t, and at equilibrium, t=∞, respectively; *D* (m^2^·s^−1^) is the diffusion coefficient and *l* is the thickness of the film. A hand-held micrometer (Hahn & Kolb, Stuttgart, Germany) was used for film thickness measurements. Five readings were taken for each sample.

#### 2.4.7. Mechanical Analysis

Tensile tests were performed using a Lloyd Instruments (Ametek Inc., Berwin, PA, USA) LR10K universal testing machine, equipped with a 100 N load cell. Rectangular specimens (15 mm·width) were used for the tests, at an initial grip separation of 100 mm and crosshead speed of 300 mm·min^−1^ along the machine direction and 200 mm·min^−1^ along the transverse direction. Average yield stress (YS), tensile stress (TS) and elongation at break (EB) were calculated from five measurements, according to ASTM D882-09 standard procedures [49].

#### 2.4.8. Antimicrobial Assays

*Micro-atmosphere diffusion antimicrobial assays*: The antibacterial activity of the films was evaluated by measuring the inhibition of *Escherichia coli* (*E. coli*, ATCC 8739) growth, by a modified micro-atmosphere diffusion method, on LB agar [50]. *E. coli* was maintained on polystyrene beads at −80 °C, and a bacterial culture was prepared by incubating one polystyrene bead in 5 mL of LB medium, for 16 h, at 37 °C, under shaking (250 rpm). Subsequently, the culture was diluted 1:100 in fresh LB medium and incubated for an additional ~1.5 h, allowing the cells to enter their logarithmic stage. When the culture reached an optical density value of 0.6, it was diluted by 1:1000 with 0.85% *w*/*w* NaCl, in distilled water, to obtain a bacterial stock solution at a concentration of 10^4^ colony forming units (CFU)·mL^−1^. Petri dishes containing LB agar were seeded with 0.1 mL of 10^4^ CFU·mL^−1^ *E. coli* stock culture, and a film sample (with an area of ~36 cm^2^) was attached, using double-sided tape, to the center of the Petri dish lid, assuring no direct contact between the film and the agar. The plates were tightly sealed with Parafilm^®^ and incubated for 12 h at 37 °C. The antibacterial potency of the films was estimated by measuring the observed inhibition zone below the films. All measurements were performed in triplicate. Neat PP and PP/HNTs films were used as reference materials.

*Micro-atmosphere diffusion antifungal assay:* The phytopathogenic, clinical and food spoilage fungi, *Alternaria alternata* (*A. alternata*), originating from the surface of a tomato, was cultured on 1% potato dextrose agar (PDA: 10 g·L^−1^; Bacto-agar: 11 g·L^−1^ in deionized water). Agar plugs (3 × 3 mm) were removed from the growing edges of a 5-day-old *A. alternata* culture and placed in the center of the Petri dish onto 1% PDA. A film sample (with an area of ~36 cm^2^) was attached to the center of the Petri dish lid, as detailed in the previous section. Subsequently, the plate was tightly sealed, inverted, and incubated for 5 days, at 25 °C, in the dark. The diameter of the growing colonies was monitored and recorded. All tests were carried out in triplicate. Neat PP and PP/HNTs films were used as reference materials.

#### 2.4.9. Molecular Dynamics Simulations

*Solubility parameter* δ is the square root of the cohesive energy density (CED, *E_coh_*). The solubility parameter is a measure of the ability of materials to dissolve each other. Cohesive energy is the energy required to break the interactions between molecules. Generally, it is measured as the heat of vaporization of a liquid. The solubility parameter calculation scheme was as follows: five amorphous cells of each single constituent (PP, HNTs and carvacrol) and the mixtures were built, using Amorphous Cell Builder. The building process was followed by geometry optimization of 5000 steps. Then, dynamic calculation was followed, using NVT ensemble (fixed temperature (298 K), fixed number of atoms and fixed volume). The dynamic stage was 500,000 fs steps long: 200,000 steps for equilibration and 300,000 for data collection every 2000 steps. Universal forcefield was used for all calculations. HNTs was represented by Kaolin molecules at approximately the same concentration as the HNTs. Enthalpies of mixing values for PP-HNTs and PP-HNTs-carvacrol systems were calculated using solubility parameters, derived from molecular dynamics simulations (MD) by the following equation (Alperstein, Knani 2013):

Δ*H_mix_abc_* = *E_coh_abc_* − (*ϕ_a_E_coh_a_* + *ϕ_b_E_coh_b_* + *ϕ_c_E_coh_c_*) (3)

where *E_coh_* is cohesion energy of the pure component or the mixture and *ϕ* is the volume fraction of each component in the mixture; *a*, *b* and *c* are the component indices.

*Radial distribution function* (RDF), also referred to as pair correlation function, gives a measure of the probability that, given the presence of an atom at the origin of an arbitrary reference frame, there will be an atom with its center located in a spherical shell of infinitesimal thickness at a distance, *r*, from the reference atom. RDF may serve as a tool to estimate intermolecular interactions. [51]. The height of the RDF frequency peak should exceed one, in order to have a significant interaction. Since the interaction potential follows the Lenard Jones potential, it is a function of 1/*r*^6^, where *r* is the interaction distance. Hence, this potential is significant only for an interaction distance of a few Angstroms, and it vanishes quickly at large separations.

## 3. Results

### 3.1. Films Morphology and Composition

**Figure 1:**
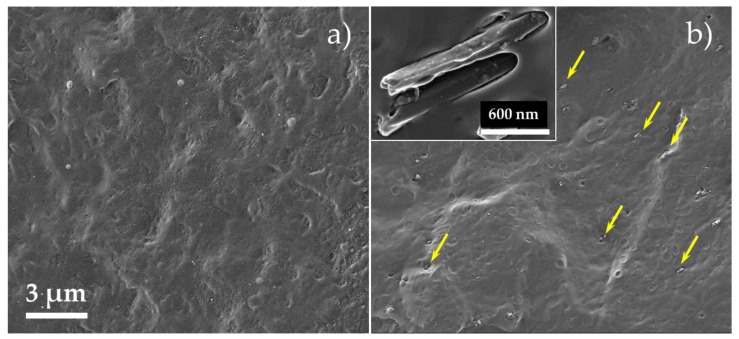
High-resolution scanning electron microscope (HR-SEM) images of cryo-fractured cross-sections of (**a**) neat PP and (**b**) PP/(HNTs-carvacrol) films. Several HNTs are marked with arrows for clarity. The HNTs are finely dispersed within the PP matrix. The inset shows a high-magnification micrograph, depicting HNTs protruding from the PP matrix. The HNTs exhibit a characteristic morphology of cylindrical tubes, with an external diameter of up to 100 nm.

Figure 1 presents HR-SEM images of a cross-sectioned PP/(HNTs-carvacrol) film and the reference neat PP film. Micrographs of PP/carvacrol and PP/HNTs films are shown in Figure S1 (Supplementary Materials). The HNTs are observed to be well-dispersed in the PP matrix, and aggregates or percolating networks are not detected. These results coincide well with previous studies reporting that HNTs are easily dispersible in polymers in comparison to other silicates, owing to the poor cohesion between the HNTs particles [52]. The inset in Figure 1b depicts a high-magnification image of two nanotubes, showing that the HNTs are coated with a thin layer of polymer, which may suggest good interactions between the PP matrix and the HNTs. The latter is further supported in the next chapter, where negative energy of mixing for PP/HNTs is calculated by molecular dynamic simulations, implying good solubility of HNTs in the PP. Yet, the few circular holes observed in Figure 1b indicate that some of the HNTs are pulled out of the polymeric matrix during the cryofracturing process. This behavior, as well as gaps between the nanotubes and the PP matrix, were observed in several studies and were related to poor interfacial interaction between pristine HNTs and PP [53].

The carvacrol concentration and distribution within the PP matrix are highly-important parameters, governing the antimicrobial and mechanical properties of the nanocomposites. The total carvacrol content in the films (i.e., post melt-compounding and film production) was determined by TGA, and the results are summarized in Table 2 (the raw thermograms are presented in Figure S2, Supplementary Materials). Carvacrol content is determined based on the mass loss of the films, occurring up to a temperature of 250 °C, ascribed to carvacrol evaporation [34,35]. For both PP/carvacrol and PP/(HNTs-carvacrol) films, the carvacrol concentration is found to be 3.1% *w*/*w*, indicating that ~80% of the initial carvacrol content is retained during the high-temperature processing steps. Previous studies have shown much lower carvacrol retention (~30–40% *w*/*w*) during PP/carvacrol film production by batch melt-compounding and subsequent compression molding [16,54]. The results obtained in present study are significantly different and may be ascribed mainly to the processing conditions, i.e., continuous vs. batch melt-compounding and film production (via cast extrusion vs. compression molding). In our case, the total calculated residence time, i.e., for both melt-compounding and cast extrusion, was 3 min. Whereas, when batch compounding was used by Ramos et al., the high-temperature processing time was notably longer (18 min) and therefore, may lead to significant losses of the volatile EO [16].

It should be noted that the entrapment of carvacrol within HNTs (prior to melt-compounding) did not affect its content within the films, while our previous studies have demonstrated that for LDPE [35] and polyamide [36] matrices, carvacrol loading into HNTs was crucial to achieve a high EO content and improved film quality. Thus, the present work indicates that the carvacrol can withstand the harsh conditions (high temperature and shearing) applied during its compounding with PP, resulting in uniform and transparent carvacrol-containing PP films. Moreover, the HNTs did not affect the thermal stability of PP, as witnessed by the similar degradation profile of the nanocomposites and the neat PP (Figure S2, Supplementary Materials). 

**Figure 2:**
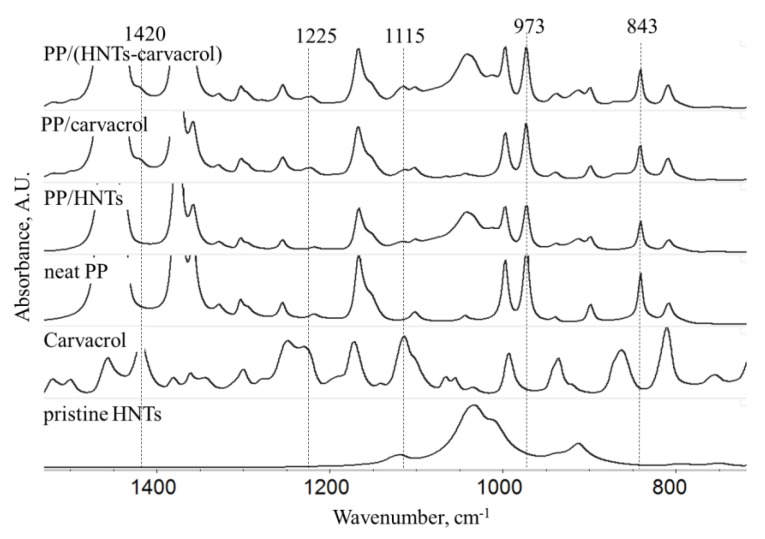
Fourier-transform infrared (FTIR) spectra (transmission mode) of neat PP, PP/HNTs, PP/carvacrol and PP/(HNTs-carvacrol) films, presenting the 1800–700 cm^−1^ region. Spectra of pristine HNTs and carvacrol are also included for comparison. The spectra of the PP/carvacrol and PP/(HNTs-carvacrol) films suggest homogeneous distribution of the carvacrol within the PP matrix. Peaks at 973 cm^−1^ and 841 cm^−1^ were used for estimation of the crystallinity in the PP films. Note that the spectrum of carvacrol is presented on a smaller absorbance scale, for clarity.

FTIR spectroscopy was used to study the composition of the films, and the results are presented in Figure 2. The latter figure depicts only the significant region in the FTIR spectra of the studied films and the full spectra are included in Figure S3 (Supplementary Materials). Spectra of pristine HNTs and carvacrol are also displayed for reference. The HNTs spectrum displays typical absorbance peaks at 3700–3600 cm^−1^ (O–H bond stretching), at 1024 and at 750 cm^−1^ (perpendicular Si–O stretching), at 900 cm^−1^ (O–H deformation), and at 796 cm^−1^ (and symmetric stretching of Si–O) [55]. The small and broad band at 3550 cm^−1^ belongs to adsorbed water [56], indicating its incomplete removal during the overnight drying process. The spectrum of neat carvacrol depicts the strongest absorption in the 900–650 cm^−1^ region, ascribed to the C–H out-of-plane vibrations of the aromatic ring. Overtone and combination bands, due to the C–H out-of-plane deformation vibrations, are observed between 2000 and 1660 cm^−1^. The absorbance in the regions 1300–1050 cm^−1^ and 850–620 cm^−1^ stems from vibrational modes of the aromatic carbon-X bond. The spectrum of the neat PP exhibits characteristic strong bands near 2950 cm^−1^, 1460 cm^−1^ and 1380 cm^−1^, and bands of medium intensity around 1155 cm^−1^ and 970 cm^−1^ [57]. The spectra of PP/HNTs, PP/carvacrol and PP/(HNTs-carvacrol) films exhibit all the major spectral elements from the individual components. Yet, some of the carvacrol-characteristic peaks, e.g., at 1180 cm^−1^ and at 805 cm^−1^, are masked by the PP bands. 

It should be emphasized that at least five different regions of each film were studied, and the obtained spectra were highly reproducible (data not shown), indicative of their homogeneous composition, further confirming results obtained by HR-SEM (Figure 1).

### 3.2. Interactions between PP, HNTs and Carvacrol by Molecular Dynamics Simulations

As the degree of compatibility between the polymer matrix and additive components greatly affects the morphology and structure of the resulting materials [6], FTIR spectroscopy was also used to investigate interactions between the nanocomposite components. When comparing the IR spectra of the films to those of the neat components (PP, HNTs and carvacrol), no evident shifts in the characteristic peaks are observed, see Figure 2. As the sensitivity of the FTIR may be insufficient to characterize such interactions [58] if they exist, we have employed molecular dynamics (MD) simulations as a tool to study the interactions between the nanocomposite components. 

**Figure 3:**
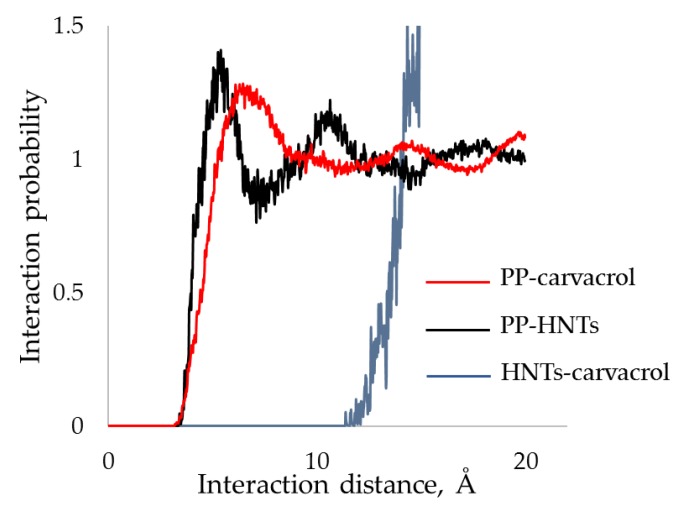
Radial distribution function for different component pairs. The interaction distance between HNTs and carvacrol is much longer compared to PP-carvacrol and PP-HNTs pairs, implying that carvacrol and HNTs only interact separately with PP, with no impact on each other.

The solubility parameters (δ) for PP, HNTs, carvacrol, and their combinations, were calculated using MD simulations and the results are summarized. In addition, we calculated the enthalpy of mixing values (Δ*H_mix_*) using Equation (3) [51], and obtained values are included in Table 3. The energy of mixing values of PP-HNTs and PP-HNTs-carvacrol are both negative and very similar, suggesting that the HNTs and carvacrol interact separately with PP, and they do not interact between themselves. To further investigate this hypothesis, we simulated the RDF for different pairs of components, and the results are presented in Figure 3. The RDF function measures the unnormalized probability to find an atom of a certain molecule at a predefined distance from another atom placed in the same space [51]. In this case, the probability of finding a PP carbon atom from a carbon atom on the carvacrol molecule, or from a silicon atom on the HNTs, is roughly the same, as the main interaction distance and the intensity are very similar for both PP-carvacrol and PP-HNTs pairs. On the other hand, the interaction distance of HNTs and carvacrol is much longer, implying that the HNTs do not interact with carvacrol. Therefore, carvacrol and HNTs only interact separately with PP, with no impact on each other, as was evident from the Δ*H_mix_* calculations (Table 3). 

It should be mentioned that the MD simulations, in this study, cannot take into consideration that carvacrol is entrapped within HNTs prior to film production. This, in turn, may somewhat reduce the predicting power of the calculations for the PP/(HNTs-carvacrol) systems. Nevertheless, the MD simulation results allow us to speculate that the interactions between carvacrol and HNTs in a PP matrix are much weaker than the interactions of carvacrol and PP. Therefore, carvacrol is stabilized during the high-temperature processing by interactions with the PP matrix, rather than by interactions with HNTs. Thus, under the conditions studied in this work, entrapment of carvacrol within HNTs does not result in a higher carvacrol content in the PP-based films.

### 3.3. Polypropylene Crystalline and Amorphous Phases (DSC, FTIR, WAXS)

The degree of crystallinity as well as the crystalline structure of semi-crystalline polymers plays a key role in determining their resulting properties [59,60]. HNTs were shown to influence the crystallization process of different semi-crystalline polymers [23]. In PP/HNTs nanocomposites, in which the HNTs content was >5% *w*/*w*, it was shown that the nanotubes can act as a nucleating agent for the alpha and beta crystalline phases, i.e. α-iPP and β-iPP [61], resulting in an increased PP crystallinity [62]. The addition of volatile compounds, such as carvacrol, was reported to either increase crystallinity by promoting diffusional mobility of the polymer chains [22] or to decrease crystallinity owing to interactions of carvacrol with the polymer matrix [16]. Herein, we have used DSC to characterize the effects of HNTs and carvacrol on the PP crystalline characteristics. Thus, the thermal behavior of the PP/(HNTs-carvacrol) nanocomposite was studied and compared to that of neat PP, PP/carvacrol and PP/HNTs films. The latter films were investigated in an attempt to elucidate the effect of the individual components, i.e., HNTs and carvacrol.

**Figure 4:**
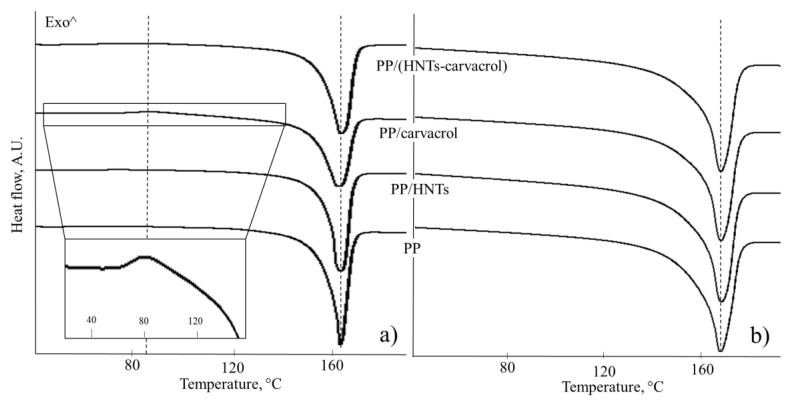
Differential scanning calorimetry (DSC) curves of the PP, PP/HNTs, PP/carvacrol and PP/(HNTs-carvacrol) films: (**a**) first heating and (**b**) second heating steps (at a rate of 10 °C·min^−1^). PP/carvacrol films exhibit solid–solid transition slightly above 80 °C (emphasized in the inset) and appear to have less ordered crystalline structure than the other films. The curves are offset along the *Y*-axis for clarity.

Figure 4 presents the DSC curves (first and second heating cycles) for the studied films and Table 4 summarizes the thermal characteristics obtained. 

The DSC curve of PP/carvacrol film features a clear exothermal peak around 90 °C during the first heating (Figure 4a, inset), which is assigned to the conversion of a mesomorphic crystalline form to an α–form, upon heating [63]. This solid–solid transition is very weak and almost undetectable for the PP/(HNTs-carvacrol) films, and is not observed for the neat PP and PP/HNTs films during the first heating, or in any of the studied films upon the second heating. The mesomorphic phase, which is also referred to as paracrystalline or smectic, is a solid phase, where the polymer chains have undergone conformational ordering, from random coils to 3/1 helices, but the helices are not densely packed. As a result, it is characterized by small-ordered clusters of parallel chains in the direction of the chain axes and lateral disorder [64]. Thus, being highly-ordered in only one direction, it has molecular ordering between amorphous and crystalline phases. The mesomorphic phase may be detected in iPP that was rapidly quenched from the melt, and is readily converted into the more stable α–form, by annealing at elevated temperatures [65]. In our case, the formation of the mesomorphic phase is facilitated by the addition of carvacrol to the neat PP.

The endothermal peaks in Figure 4 at around 167 °C are attributed to the melting of α-crystals of the PP [61]. Figure 4a shows that the fusion peaks of PP (first heating cycle) for the carvacrol-containing films (both PP/carvacrol and PP/(HNTs-carvacrol)) are slightly broader than those for the neat PP and PP/HNTs films, suggesting that carvacrol induces heterogeneity in the PP crystalline structure. The degree of crystallinity, calculated using Equation (1), for the first heating cycle is similar for all studied films. However, the endothermal event of PP melting, between 130 and 170 °C (Figure 4a), probably masks the endothermal event, which is ascribed to the evaporation of carvacrol from the films. This suggestion is supported by the TGA thermograms (see Figure S2), where carvacrol is lost from the PP films in this temperature range. Thus, the crystallinity calculated for the first heating cycle, for the carvacrol-containing PP films, is an overestimation and does not reflect the actual crystallinity in the as-produced PP/carvacrol and PP/(HNTs-carvacrol) films. In contrast, the degree of crystallinity calculated from the second heating step is more accurate, as most of the carvacrol is lost during the first heating, and these values are found to be similar for all studied films, see Table 3. Moreover, the shape of these melting peaks is similar for all studied systems (Figure 4b) as well as the melting temperature values (~167 °C), which are comparable. Thus, these results indicate that the effect of HNTs (at low concentrations of 2% *w*/*w*) on PP crystallinity is minor and in agreement with previous studies [61], where HNTs were found to influence the PP crystalline structure at concentrations higher than 5% *w*/*w*. 

As the overlapping exothermal events of the PP melting and carvacrol evaporation, during the first heating step, prevent accurate determination of the degree of crystallinity in the as-produced PP/carvacrol and PP/(HNTs-carvacrol) films, we have employed both IR spectroscopy and WAXS studies. These techniques are non-destructive and are performed at a room temperature, eliminating artefacts caused by specimen instability, as in the case of DSC, where heating triggers fast carvacrol release from the studied specimen during the measurement [66].

**Figure 5:**
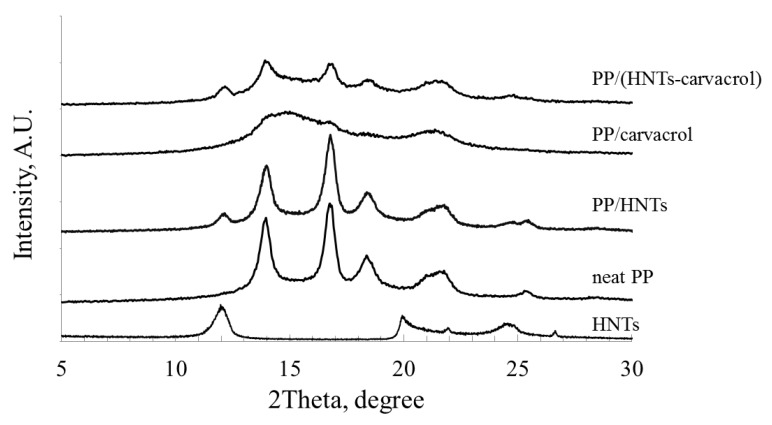
WAXS diffractograms of neat HNTs and all films. The diffractograms are offset along the *Y*-axis, for clarity.

From the IR spectra of the films, presented in Figure 2, we have extracted the absorbance values at 841 and 973 cm^−1^ and calculated the ratio between these bands (A_841_/A_973_). The latter ratio was shown to reflect the degree of crystallinity of PP films [67]. Table 4 summarizes the calculated A_841_/A_973_ ratio for all films. The PP/HNTs nanocomposites exhibited the highest A_841_/A_973_ value, greater than those of neat PP films, suggesting a nucleating effect of the HNTs. The lowest A_841_/A_973_ value was obtained for the PP/carvacrol films, indicating that the addition of carvacrol to the neat PP reduces the PP crystallinity, in comparison to neat PP. The similar values for the A_841_/A_973_ ratio, determined for both neat PP and PP/(HNTs-carvacrol) systems, suggest that the films exhibit a similar degree of crystallinity, while their crystalline structures may differ. To further elucidate the effects of carvacrol and HNTs on the degree of crystallinity and on the crystalline structure of PP, and to validate the results obtained by the DSC and IR spectroscopy, the films were studied by WAXS (Figure 5). 

The WAXS diffractogram for the neat PP exhibits characteristic peaks at 2Θ of 14.0° (6.3 Å, 110 plane), 16.9° (5.2 Å, 040 plane), 18.5° (4.7 Å, 130 plane) and 21.7° (4.1 Å, 111 plane), in agreement with previous studies [61]. The PP/HNTs diffractogram combines all the major PP-related peaks with the HNTs characteristic peaks, at 2Θ of 12.0° (7.4 Å, 001 plane), and 24.5° (3.6 Å, 002 plane) [23]. In contrast, in the PP/carvacrol system, the PP-characteristic peaks completely disappear, and only broad and diffuse halos (at 2Θ of 15° and 22°) are observed. This diffractogram was previously shown to characterize the mesomorphic form of iPP homopolymer [65]. Thus, the WAXS analysis for the PP/carvacrol films confirms the DSC result (Figure 4, inset), where transition of the mesomorphic crystalline form to an α–form upon heating is observed. In the PP/(HNTs-carvacrol) nanocomposites, the major diffraction features of both PP and HNTs are superimposed onto the mesophase halos (Figure 5).

The bands from the second order of diffraction are usually observed in highly-ordered samples only, while they tend to disappear in poorly-ordered polymers. The diffractograms of the neat PP and PP/HNTs films feature a broad band at ~28°, ascribed to the second order diffraction from the 110 plane of the PP (corresponding to 2Θ of 14°). This band is barely observable for the PP/(HNTs-carvacrol) system, and cannot detected for the PP/carvacrol films, thus indicating that the degree of molecular order in the PP/carvacrol films is significantly lower than in PP/(HNTs-carvacrol) nanocomposites.

The degree of crystallinity for all films was determined from the WAXS diffractograms after their careful deconvolution, and the results are summarized in Table 4. The WAXS-based degrees of crystallinity values are in excellent agreement with the IR studies, where the lowest crystallinity is observed for the PP/carvacrol system. The PP/HNTs film crystallinity is slightly higher than that of neat PP, ascribed to the HNTs nucleating effect, and the PP/(HNTs-carvacrol) nanocomposites demonstrate crystallinity similar to the neat PP films. It may be suggested that in the PP/carvacrol films, carvacrol interference with the PP crystalline structure may lead to the formation of a disordered mesomorphic solid state, while the loading of carvacrol upon HNTs helps to preserve a stable and ordered crystalline structure. 

The effects of the HNTs and carvacrol on the PP amorphous phase in the nanocomposite films were studied by DSC, and the glass transition temperature (*T_g_*) values are summarized in (Table 4). Note that to measure the *T_g_*, the films were quenched, and a high heating rate of 50 °C·min^−1^ was used. Previous work by Persico et al. [22] has demonstrated the plasticization effect of carvacrol on LDPE matrices. However, the effects of carvacrol and/or HNTs on the PP amorphous phase were not studied, to the best of our knowledge. The displayed *T_g_* values for both PP/carvacrol and PP/(HNTs-carvacrol) films are significantly lower (by 7 °C and 6 °C, respectively) in comparison to neat PP. These results reveal the carvacrol plasticization effect on the PP matrix. It is worth mentioning that neat PP and PP/HNTs films exhibit a similar *T_g_* (0 °C) [63]. This result indicates that for the studied concentration, HNTs presence does not affect the amorphous phase characteristics.

### 3.4. Mechanical Properties

The structure and orientation of the crystalline and amorphous phases greatly affect the mechanical properties of the polymer [59]. To elucidate the effects of carvacrol and HNTs on the mechanical properties of the cast films, tensile tests were performed, and Table 5 summarizes the results of these studies for all films in both machine and transverse directions.

The addition of HNTs to the PP matrix was not found to affect its mechanical properties. Minor, statistically insignificant, changes in the tensile stress at break (TS) were observed, aligning with our DSC and WAXS results, and with other reports [42], where HNTs had no or only a small influence on the crystallinity and mechanical properties of the PP. The addition of carvacrol to the PP matrix resulted in a profound decrease in the yield stress (YS) and an increase in the elongation at break (EB) values, in both directions. This behavior is attributed to the plasticizing effect of carvacrol, in agreement with the DSC results described in the previous section, see Table 4. The latter revealed that the carvacrol-containing PP films exhibit lower *T_g_* values (by ~7 °C) in comparison to neat PP. Our results are also consistent with earlier studies, where carvacrol was reported to increase EB in PP [16] and LDPE [22,68]. Furthermore, carvacrol addition also reduced the characteristic anisotropy in the mechanical properties of PP cast films [59], specifically, the values of EB. This observation coincides with the WAXS results (Figure 5), in which the PP/carvacrol films displayed the lowest degree of crystalline order.

The PP/(HNTs-carvacrol) nanocomposites exhibited inferior mechanical properties, in terms of YS and TS values, in comparison to the neat PP and PP/HNTs films. This behavior is ascribed to the plasticizing effect of the “free” carvacrol (carvacrol fraction which is dissolved in the amorphous phase and is not entrapped within the HNTs). Yet, these values are higher than those measured for the PP/carvacrol system, which can be ascribed to complex role of the HNTs in the nanocomposites. First, the HNTs serve as nanocarriers of carvacrol—its concentration in the PP matrix is effectively lower than in the corresponding PP/carvacrol films [69]. Second, the HNTs act as a nucleating agent in the nanocomposites, leading to a higher crystallinity. The degree of crystallinity of the PP/(HNTs-carvacrol) system is comparable to that of neat PP and PP/HNTs films (see Table 4) resulting in improved YS and TS values. Noteworthy are the superior EB values, in both machine and transverse directions, exhibited by the nanocomposites and the isotropic behavior of the films.

### 3.5. Release Studies and Antimicrobial Activity

The release kinetics of the volatile antimicrobial agent from the film is crucial for determining its antimicrobial performance and potential applicability as a packaging material. Moreover, the ability to control the release kinetics is desired for tailoring the material’s properties for a specific application or condition [6]. TGA was used to characterize the carvacrol release from the different films, by monitoring their weight loss over time, at a constant temperature (60 °C) [35,70], and Table 6 presents the calculated diffusion coefficient values. The results show that the diffusivity of carvacrol in the PP/(HNTs-carvacrol) films is lower by ~30%, in comparison to the corresponding PP/carvacrol system, leading to sustained release of the carvacrol from the nanocomposite. The latter is ascribed to the role of HNTs as: (i) nanocarriers, where the release of carvacrol from the PP/(HNTs-carvacrol) films is determined not only by the diffusion coefficient of carvacrol in the polymer matrix, but also by its out-diffusion from HNTs’s lumen and its desorption rate from HNTs’s surface [71]; (ii) nano-scale fillers, forming a tortuous diffusion path in the PP matrix [72,73]; (iii) a nucleating agent, increasing the crystallinity and facilitating the formation of more ordered crystallites (in comparison to the PP/carvacrol system).

**Figure 6:**
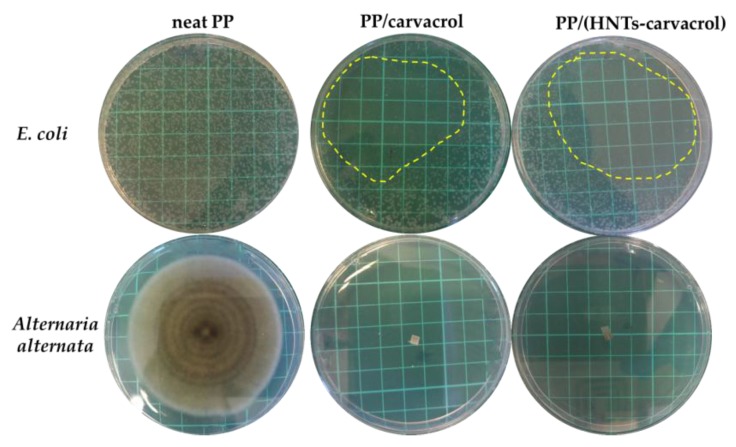
Antimicrobial effect of neat PP, PP/carvacrol and PP/(HNTs-carvacrol) films exhibited in the micro-atmosphere diffusion assays, i.e., without direct contact between the studied films and the microbial cultures. **Top panel**: Petri dishes containing the *E. coli* after incubation with the films for 12 h, at 37 °C (the margins of the inhibition zone are marked for clarity). **Bottom panel**: Petri dishes containing *A. alternata* following seven days of incubation, at 25 °C, in the dark.

To evaluate the biofunctionality of the carvacrol in the films, in terms of its antimicrobial activity, we studied the effect of the carvacrol-containing films on the growth of common bacterial and fungal microorganisms. The gram-negative *Escherichia coli* (*E. coli*) was chosen as a model bacterial species, as some of its strains are widely-spread foodborne pathogens. The model fungus is the phytopathogenic *Alternaria alternata* (*A. alternate*), which was chosen owing to its clinical relevance and its significant implications for food spoilage as well as food and feed safety [74]. Figure 6 depicts characteristic images of Petri dishes after the exposure of the two model microorganisms to the different films. Both PP/carvacrol and PP/(HNTs-carvacrol) films exhibit a profound inhibition of *E. coli* growth, as most of the dish area is observed to be colony-free, see Figure 6 (upper panel). The carvacrol-containing films also display a strong antifungal activity, completely arresting hyphal growth and sporulation of *A. alternata*. Whereas, for the reference neat PP and PP/HNTs (data not shown) films, no effect on bacterial and fungal development is observed. Thus, the high antimicrobial activity of carvacrol, which is related to its ability to damage the integrity of the cell membrane [75] and interfere with the metabolic activity of the cell [76], is retained in the films, despite their processing. 

## 4. Conclusions

The present work investigated the composition-structure-property relationships in carvacrol-containing PP films, produced by incorporating pure carvacrol and carvacrol loaded into HNTs, followed by melt-compounding and cast extrusion. Molecular dynamics simulations, performed for the first time for the PP-HNTs-carvacrol systems, revealed strong interactions between the PP matrix and carvacrol. These interactions play a role in retaining the highly-volatile EO within the PP matrix during processing at elevated temperatures. Indeed, both PP/carvacrol and PP/(HNTs-carvacrol) films exhibited relatively low losses of carvacrol—~20% *w*/*w* from the initial amount—during compounding and cast extrusion. This behavior is dramatically different than that observed for carvacrol-containing LDPE [34,35] and PA [36] systems, in which HNTs act as “active” carriers, protecting the carvacrol during high-temperatures. Yet, in the PP system, the HNTs were found to affect the properties of the PP/(HNTs-carvacrol) films, in terms of their structure and resulting mechanical properties and carvacrol release profiles. Carvacrol, acting as a plasticizer, interferes with the PP crystalline structure and facilitates the formation of a disordered mesomorphic crystalline state, which leads to inferior mechanical properties and faster carvacrol release from the films. Thus, the use of carvacrol-loaded HNTs diminishes this effect, due to the HNTs’ multifunctional action as nucleating agents, nanoscale fillers and efficient nanocarriers for carvacrol. Both PP/carvacrol and PP/(HNTs-carvacrol) films exhibit excellent antibacterial and antifungal activities in the vapor phase against model microorganisms (*E. coli* and *A. alternata*). Thus, the final properties of the films can be controlled by the careful design of the film composition, and such antimicrobial polymer systems can be potentially used for numerous applications—food, medical, personal care and hygiene—where tailor-made materials with tunable antimicrobial and mechanical properties can be designed.

## Figures and Tables

**Table 1:** Composition of studied films.

Film Name	Composition (% *w*/*w*)		
	PP	HNTs	Carvacrol
PP	100	0	0
PP/HNTs	98	2	0
PP/carvacrol	96	0	4
PP/(HNTs-carvacrol)	94	2	4

PP—polypropylene; HNTs—Halloysite nanotubes.

**Table 2:** Carvacrol content in different films measured by thermal gravimetric analysis (TGA).

Polymer Films	Pre-Processing Content of Carvacrol, % *w*/*w*	Post-Processing Content of Carvacrol by TGA, % *w*/*w*
PP	None	0.0 ± 0.0
PP/HNTs	None	0.0 ± 0.0
PP/carvacrol	4	3.1 ± 0.1
PP/(HNTs-carvacrol)	4	3.1 ± 0.1

**Table 3:** Solubility parameters of PP, HNTs and carvacrol and their enthalpy of mixing, calculated using molecular dynamics simulation.

Materials and Their Combinations	δ, MPa^0.5^	Δ*H_mix_*, MJ·m^−3^
PP	20.05	
Carvacrol	23.36	
HNTs	23.77	
PP-HNTs	17.36	−107
PP-HNTs-carvacrol	17.05	−103

δ—Solubility parameter; Δ*H_mix_*—Enthalpy of mixing.

**Table 4:** Melting, crystallization and glass transition temperatures obtained using DSC; and the degree of crystallinity evaluated, using DSC, FTIR and wide angle X-ray scattering (WAXS) for different films (the number in parenthesis indicate standard deviations obtained from at least three repetitions).

Polymer Film	DSC						FTIR	WAXS
	*T_m_*_1_	*T_m_*_2_	*T_c_*	*T_g_*_(*o*)_	*c_m_*_1_ (%)	*c_m_*_2_ (%)	A_841_/A_973_	*c* (%)
PP	167 (1)	166 (0)	123 (1)	0 (0)	37 (0)	44 (1)	0.54 (0.00)	46 (1)
PP/HNTs	168 (1)	167 (0)	123 (0)	0 (0)	38 (1)	44 (1)	0.57 (0.00)	48 (1)
PP/carvacrol	168 (0)	166 (0)	121 (0)	−7 (0)	39 (0)	43 (1)	0.48 (0.00)	41 (1)
PP/(HNTs-carvacrol)	167 (1)	166 (0)	122 (0)	−6 (0)	40 (1)	43 (1)	0.54 (0.00)	46 (1)

**Table 5:** Mechanical properties of the films along machine and transverse directions (the numbers in parenthesis indicate standard deviations obtained from at least five repetitions).

Samples	Along Machine Direction			Along Transverse Direction		
	Yield Stress, MPa	Tensile Stress at Break, MPa	Elongation at Break, %	Yield Stress, MPa	Tensile Stress at Break, MPa	Elongation at Break, %
PP	33 (1)	49 (2)	605 (34)	32 (1)	31 (1)	8 (1)
PP/HNTs	32 (1)	54 (6)	607 (50)	29 (1)	28 (2)	5 (1)
PP/carvacrol	17 (1)	48 (3)	663 (23)	17 (1)	25 (4)	499 (59)
PP/(HNTs-carvacrol)	24 (1)	not observed	>700	23 (1)	33 (1)	643 (28)

**Table 6:** Calculated diffusivity values for carvacrol from various films, determined by fitting the mathematical model for short-time diffusion-limited desorption from a polymer film surface to the release profile of carvacrol from the films measured by TGA (the numbers in parenthesis indicate standard deviations obtained from at least three independent experiments).

Polymer Film	Carvacrol Diffusivity, ×10^13^·m^2^·s^−1^
PP/carvacrol	9.42 (0.82)
PP/(HNTs-carvacrol)	6.99 (0.61)

## Supplementary Materials

The following are available online at www.mdpi.com/2073-4360/10/1/79/s1, Figure S1: HR-SEM images of cryo-fractured cross-section of (a) PP/carvacrol and (b) PP/HNTs films. Several HNTs are marked with arrows for clarity, Figure S2: TGA thermograms of neat PP; PP/HNTs; PP/carvacrol; and PP/(HNTs-carvacrol) nanocomposite films over the entire temperature region studied, Figure S3: Full FTIR spectra (transmission mode) of pristine HNTs, carvacrol, neat PP, PP/HNTs, PP/carvacrol and PP/(HNTs-carvacrol) films.
