# Supplementary material for: Antimicrobial Carvacrol-Containing Polypropylene Films: Composition, Structure and Function
Source: Polymers (Basel). 2018 Jan 16;10(1):79. doi: 10.3390/polym10010079 (PMC6415180; doi:10.3390/polym10010079)
Supplement: Supplementary file 1 [file polymers-10-00079-s001.pdf]

Supplementary Materials

Antimicrobial Carvacrol-Containing Polypropylene Films: Composition, Structure and Function

Max Krepker [1], Ofer Prinz-Setter [1], Rotem Shemesh [2], Anita Vaxman [2], David Alperstein [3] and Ester Segal [1,4,*]

[1] Department of Biotechnology and Food Engineering, Technion—Israel Institute of Technology, Haifa 3200003, Israel; maks@campus.technion.ac.il (M.K.); oferp@campus.technion.ac.il (O.P.-S.)

[2] Carmel Olefins Ltd., P.O. Box 1468, Haifa 31014, Israel; Srotem@bazan.co.il (R.S.); Vaxman.anita@gmail.com (A.V.)

[3] Department of Mechanical Engineering, Ort Braude College, P.O. Box 78, Karmiel 2161002, Israel; davida@braude.ac.il

[4] The Russell Berrie Nanotechnology Institute, Technion—Israel Institute of Technology, Haifa 3200003, Israel

* Correspondence: esegal@technion.ac.il

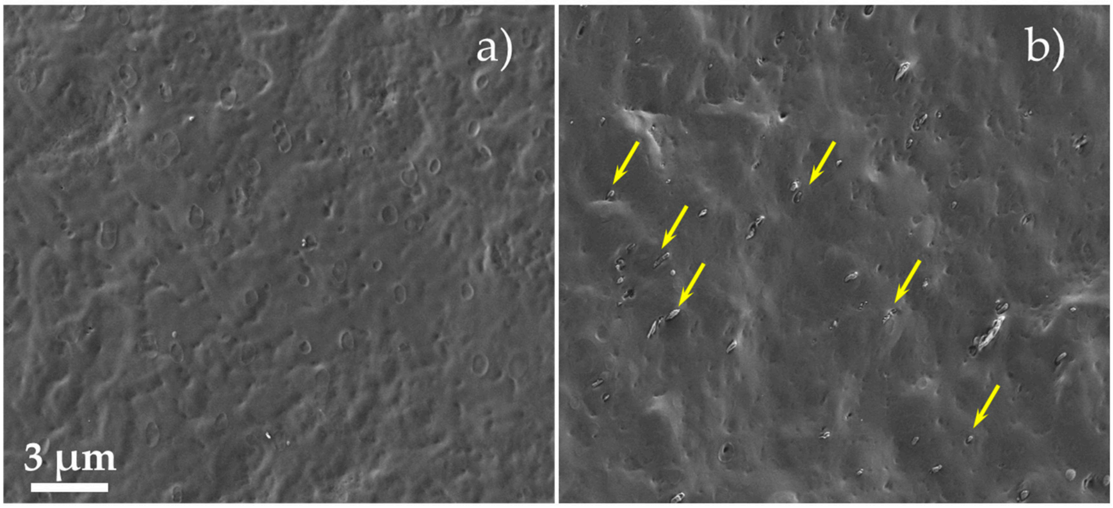

Figure S1. HR-SEM images of cryo-fractured cross-section of a) PP/carvacrol and b) PP/HNTs films. Several HNTs are marked with arrows for clarity.

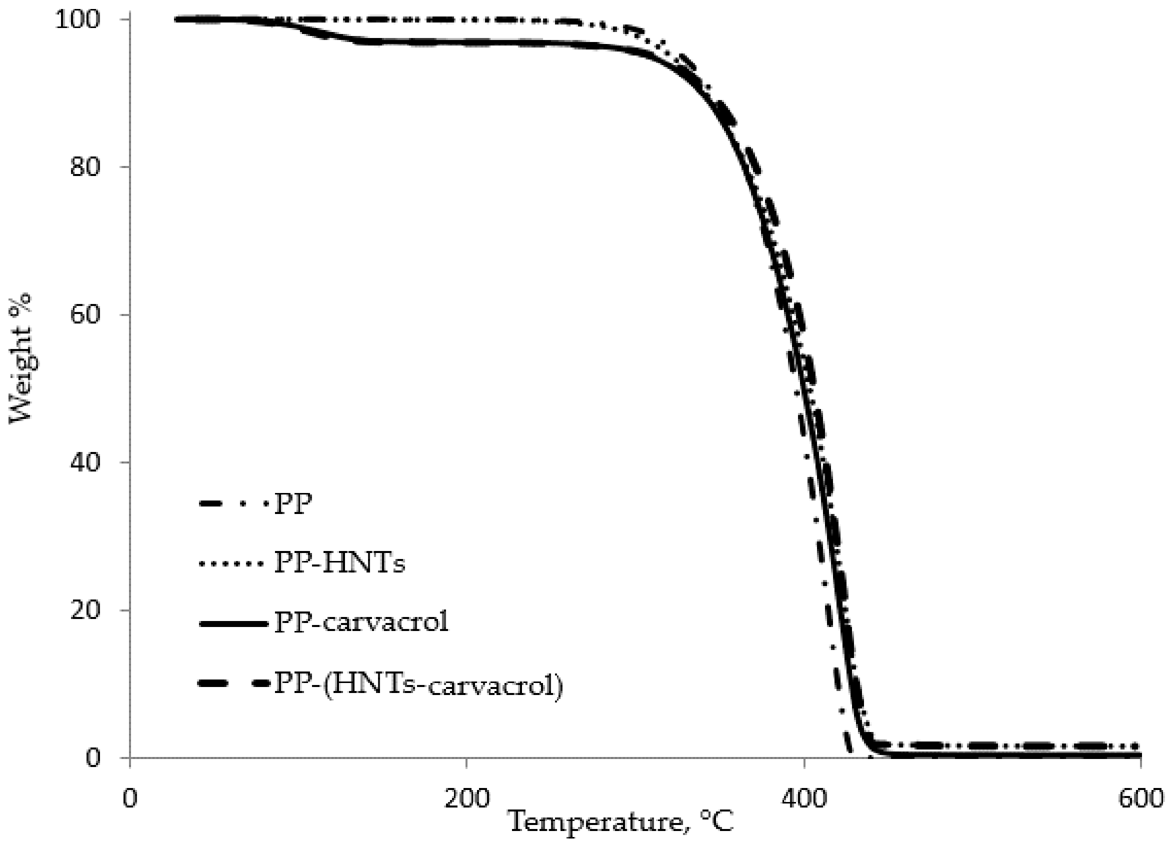

Figure S2. TGA thermograms of neat PP; PP/HNT; PP/carvacrol; and PP/(HNTs-carvacrol) nanocomposite films over the entire temperature region studied.

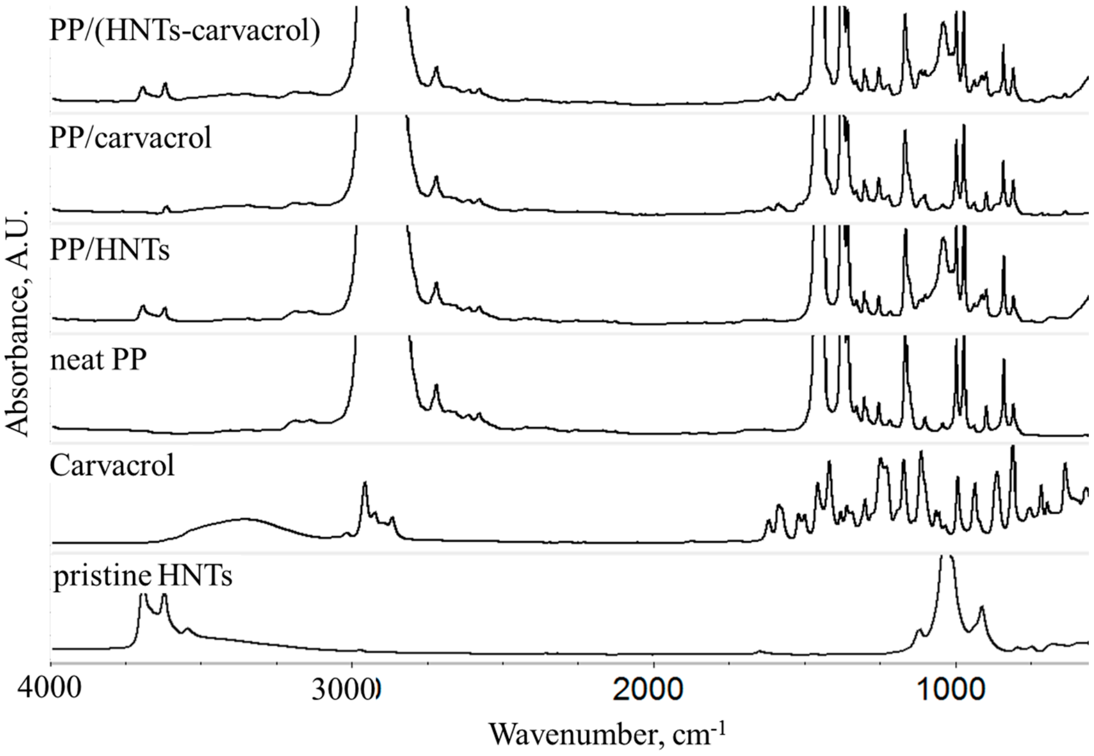

Figure S3. Full FTIR spectra (transmission mode) of pristine HNTs, CV, neat PP, PP/HNTs, PP/carvacrol and PP/(HNTs-carvacrol) films.
